# Predictors of positive patient-reported outcomes from ‘Early Intervention in Psychosis’: a national cross-sectional study

**DOI:** 10.1136/bmjment-2023-300716

**Published:** 2023-08-04

**Authors:** Ryan Williams, Aimee Morris, Veenu Gupta, Ed Penington, Alexis E Cullen, Alan Quirk, Paul French, Belinda Lennox, Alex Bottle, Mike J Crawford

**Affiliations:** 1 Department of Brain Sciences, Imperial College London, London, UK; 2 College Centre for Quality Improvement, Royal College of Psychiatrists, London, UK; 3 Department of Psychological Sciences, University of Liverpool, Liverpool, UK; 4 Department of Psychiatry, University of Oxford, Oxford, UK; 5 Department of Psychosis Studies, King's College London, London, UK; 6 Department of Clinical Neuroscience, Karolinska Institutet, Stockholm, Sweden; 7 Department of Psychology, Manchester Metropolitan University, Manchester, UK; 8 School of Public Health, Imperial College London, London, UK

**Keywords:** Schizophrenia & psychotic disorders, Adult psychiatry

## Abstract

**Background:**

The components of care delivered by Early Intervention in Psychosis (EIP) services vary, but the impact on patient experience is unknown.

**Objective:**

To investigate associations between components of care provided by EIP services in England and patient-reported outcomes.

**Methods:**

2374 patients from EIP services in England were surveyed during the National Clinical Audit of Psychosis. Participants were asked about the care they received, and completed the ‘Patient Global Impressions’ Scale (rating whether their mental health had improved), and ‘Friends and Family Test’ (rating whether they would recommend their service). Information about service structure was obtained from service providers. We analysed associations between outcomes and components of care using multilevel regression.

**Findings:**

The majority of participants were likely to recommend the treatment they had received (89.8%), and felt that their mental health had improved (89.0%). Participants from services where care coordinators had larger case loads were less likely to recommend their care. Participants were more likely to recommend their care if they had been offered cognitive behavioural therapy for psychosis, family therapy or targeted interventions for carers. Participants were more likely to report that their mental health had improved if they had been offered cognitive behavioural therapy for psychosis or targeted interventions for carers.

**Conclusions:**

Specific components of EIP care were associated with improved patient reported outcomes. Psychosocial interventions and carer support may be particularly important in optimising outcomes for patients.

**Clinical implications:**

These findings emphasise the need for small case load sizes and comprehensive packages of treatment in EIP services.

**WHAT IS ALREADY KNOWN ON THIS TOPIC**

- EIP input has been associated with a range of benefits compared with treatment as usual.
- However, studies of patient experience are lacking.
- No studies have examined associations between patient experience and specific components of EIP care.

**WHAT THIS STUDY ADDS**

- Specific components of EIP care—smaller care coordinator case loads, and provision of CBT, family therapy and carer interventions—are associated with improved patient experience.

**HOW THIS STUDY MIGHT AFFECT RESEARCH, PRACTICE OR POLICY**

- These findings should guide EIP service implementation. Clinicians working in EIP settings should be aware of components of care, as well as patient demographic factors, which may be associated with improved experience and outcome of treatment.

## Background

Psychotic disorders are severe mental health conditions with wide-ranging consequences including poor physical health and premature mortality, alongside difficulties with education, employment and relationships.^1 2^ Early Intervention in Psychosis (EIP) services are specialised community-based multidisciplinary mental health teams that work selectively with people in the early stages of psychosis.^3 4^ Meta-analytical studies have demonstrated superior outcomes for people who receive EIP team input compared with ‘treatment as usual’.^5^ EIP services appear to be cost-effective,^6^ and this model of treatment has been widely implemented in the UK^7^ and internationally.^8^

Despite the enthusiasm for EIP services, important unanswered questions remain regarding processes of EIP care. A recent review found a lack of meaningful input from patients and their families in the evaluation of EIP services, and called for greater utilisation of patient-reported outcome measures (PROMs).^9^ Patient-reported outcomes are intrinsically important, and are associated with engagement and other clinically important outcomes.^10^

It is also not currently clear which components of EIP services contribute to their observed benefits. Guidelines for EIP implementation specify that they should offer a package of interventions including specialised psychological therapies, tailored medication regimens and dedicated support for carers.^11 12^ They also include specific structural and staffing criteria, stressing the importance of small care-coordinator case loads to facilitate intensive case management.^11^ Significant variation in service structure and adherence to these guidelines has been found, even between services in the same region.^13^ Studies attempting to link specific components of EIP care with positive outcomes have been inconclusive.^14^

### Objective

In this study, we aimed to investigate whether specific components of EIP services (treatments offered, case load size) were associated with improved patient experience and self-reported improvements in mental health.

## Methods

Data for this study were collected from a patient survey and service-level ‘contextual questionnaire’ as part of the ‘National Clinical Audit of Psychosis’ (NCAP) between July and October 2019. The NCAP is a multiphase quality improvement programme conducted by the UK Royal College of Psychiatrists.^15^

In 2019, the NCAP included a survey of people using EIP services throughout England. This survey included PROMs relating to experience of care and improvement in mental health, as well as questions about the specific components of care and treatments received by each participant. During the same audit phase, linked contextual data on EIP service workforces and case load sizes were collected directly from the services themselves. A detailed account of the methods used in the NCAP have been published elsewhere.^16^

All EIP services funded by the National Health Service (NHS) in England were invited to participate in the NCAP (n=155 services). Participating teams sent surveys to a random sample of 150 eligible patients. Patients were eligible for inclusion if they met the following criteria: age 14–65 years; on the case load for >6 months at the time of the survey; an established diagnosis of a ‘first episode’ of any primary psychotic disorder (including affective, non-affective and substance-related psychoses). They were excluded (ie, not sent a survey) if they had a primary diagnosis of psychosis due to an ‘organic’ cause.

Participants had the option of completing either a paper or a web-based version of the survey. In order to minimise response bias, this included clear instructions that the survey was confidential. Consent was implied by response to the survey.

The National Research Ethics Service and the Ethics and Confidentiality Committee of the National Information Governance Board were consulted and advised that formal ethical approval was not required to undertake secondary analysis of anonymised NCAP data for service improvement purposes. The authors assert that all procedures contributing to this work comply with the ethical standards of the relevant national and institutional committees on human experimentation and with the Helsinki Declaration of 1975, as revised in 2008.

### Main outcome measure and covariates

Our primary outcome measure was the ‘Friends and Family Test’^17^—a single item measure of patient experience that has been widely used across the NHS, including in the assessment of treatments for people with psychosis.^18^ Respondents were asked, ‘how likely are you to recommend your EIP worker/team to friends and family if they needed similar care or treatment?’ using a 5-point scale (extremely likely, likely, neutral, unlikely, extremely unlikely).

As a secondary outcome measure, we used the ‘Patient Global Impressions – Improvement’ (PGI-I), another validated measure of patient experience used to assess treatment outcomes of people with severe mental illness.^19^ Respondents were asked, ‘overall, has your mental health improved or got worse since you have been under the care of your EIP Team?’, again using a five-point scale (much improved, a little improved, no change, a little worse, much worse).

The patient survey also included a series of questions about whether respondents had been offered specific treatments (antipsychotic medication, cognitive behavioural therapy for psychosis or ‘CBTp’, family therapy, carer support) as well as questions about their experience of specific aspects of their care.

Finally, respondents were asked to indicate their age, gender and ethnic background from a range of categorical options. It was not possible to collect demographic data from people who did not respond to the survey, but these were available from a parallel audit of clinical records (undertaken for the entire sample of eligible participants who had been sent a survey) that was conducted as part of the NCAP at the same time.

The service-level contextual questionnaire asked services to indicate their total case load size and the number of full-time equivalent care coordinators employed at their service. From this information, we were able to calculate the mean case load size of a care coordinator at each service.

### Statistical methods

All analyses used ‘R’ V.4.2.2. Initially, we calculated the proportions of people who were likely to recommend their treatment and who felt that their mental health had improved (our primary and secondary outcome measures). We converted responses on the 5-point scales to three-level variables as we felt that these levels were more clinically relevant. For the Friends and Families Test, these levels indicated whether overall people were likely to recommend their care (combining ‘extremely likely’ and ‘likely’), neutral or unlikely (combining ‘extremely unlikely’ and ‘unlikely’). For the PGI-I, these indicated whether overall people felt their mental health had improved (combining ‘much improved’ and ‘a little improved), remained unchanged or deteriorated (combining ‘much worse’ and ‘a little worse’). The association between these outcome measures was examined using ‘Cramér’s V’ statistic.

We calculated descriptive statistics for exposures and outcomes, using appropriate measures to describe central tendency and spread for care coordinator case load size, and contingency tables for categorical variables. Following a priori discussions between coauthors with clinical expertise in this area, we created a map of theoretical associations between variables to be tested before building regression models.

We examined unadjusted associations between primary/secondary outcomes and exposures (care coordinator case load size and treatments received) using ordinal logistic regression (‘ordinal’ package in R). We also examined unadjusted associations between exposure and outcome variables and potential confounders (covariates)—respondent demographic characteristics.

Finally, we examined the association between our exposure variables (care coordinator case load and treatments received) and primary and secondary outcome variables (Friends and Family Test and PGI-I, respectively), adjusting for covariate effects. We adjusted for clustering using multilevel statistical methods (mixed-effects ordinal logistic regression), and evaluated model assumptions using Variance Inflation Factor and Brant tests (‘car’ and ‘brant’ packages in R, respectively).

Missing data varied from 0.80% to 6.71% across variables. The proportion of missing values for our primary and secondary outcomes specifically was low (3.07% and 0.84%, respectively).

For each variable, we identified those with missing and those with available data. We then compared the results of these two groups for all other variables where both had available data. In each case, there were no statistically significant differences between individuals with available and missing data, suggesting that missingness was not driven by systemic factors relating to exposures or outcomes and data were missing ‘completely at random’ (eg, individuals with missing data for each exposure variable were similarly likely to recommend their care and report improved mental health compared with those with available data). While it is important to acknowledge that this assumption cannot be definitively proven and potential biases may always be introduced by missing data, this approach indicates that the likelihood of such bias in our findings is low.

### Findings

All eligible EIP teams (n=155) submitted data for the contextual questionnaire, with 152 teams providing data for the patient survey (three unable to send out the survey due to organisational issues). Surveys were received from 2374 respondents (18% response rate). Demographic characteristics of respondents and the entire NCAP sample are summarised in table 1. People who responded to the survey were more likely to be white and female than non-responders.

**Table 1:** Demographic characteristics of study participants and comparative data from case note review

Demographic characteristics	Study sample N (%)	Total NCAP sample from case note review N (%)
**Age, years**		
<19	102 (4.4)	Mean age=32.11 SD=11.05
19–25	670 (28.8)	
25–35	667 (28.7)	
35–50	531 (22.8)	
>50	356 (15.3)	
**Ethnicity**		
White	1527 (66.3)	6766 (64)
Black/black British	243 (10.6)	1356 (13)
Asian/Asian British	232 (10.1)	1286 (12)
Mixed	119 (5.2)	421 (4)
Other	181 (7.9)	731 (7)
**Gender**		
Female	1086 (47.4)	4082 (39)
Male	1185 (51.7)	6468 (61)
Other	21 (0.9)*	10 (<1)*

*Note that the disparity here (more people of non-binary gender in the study sample despite this being a subset of the total NCAP sample) is likely due to difficulties in correctly classifying gender from case note review alone, as compared with self-report.

NCAP, National Clinical Audit of Psychosis.

Of the people, 89.8% (n=2050) were ‘likely’ to recommend the treatment that they had received, 6.2% (n=142) were ‘neutral’ and 4.0% (n=91) were ‘unlikely’; 89.0% (n=2091) felt that their mental health had improved since they had been under the care of their EIP team, 7.1% (n=167) felt that there had been no change in their mental health and 3.9% (n=92) felt that their mental health had deteriorated. These two outcomes demonstrated a weak to moderate degree of association (Cramér’s V=0.247).

The mean care coordinator case load size of a participating EIP team was 17.4 patients, with a maximum of 35.4 and a minimum of 7.5 (SD 4.7). Frequencies and proportions of participants who reported being offered specific treatments as part of their EIP care varied considerably, from 94.7% (n=2226) for antipsychotic medication to 27.1% (n=598) for family therapy. Full breakdowns are presented in tables 2 and 3.

**Table 2:** Associations between clinical/demographic variables and likelihood of recommending care

Component of care		Likely to recommend	Neutral to recommend	Unlikely to recommend	Unadjusted OR (likely vs neutral/unlikely)	Adjusted† OR (likely vs neutral/unlikely)
Care coordinator case load size (mean)		17.0	18.0	18.4	0.96 (0.93 to 0.99)**	0.96 (0.93 to 0.98)^*^
Offered CBTp	No	989 (87.2)	82 (7.2)	63 (5.6)	Ref	Ref
	Yes	985 (92.5)	56 (5.3)	24 (2.3)	1.83 (1.38 to 2.44)***	1.64 (1.13 to 2.36)**
Offered family therapy	No	1362 (87.8)	115 (7.4)	74 (4.8)	Ref	Ref
	Yes	552 (94.9)	20 (3.4)	10 (1.7)	2.56 (1.75 to 3.88)***	1.72 (1.05 to 2.82)^*^
Offered carer intervention	No	403 (80.6)	48 (9.6)	49 (9.8)	Ref	Ref
	Yes	1283 (93.6)	59 (4.3)	29 (2.1)	3.58 (2.63 to 4.89)***	3.82 (2.64 to 5.52)***
Offered antipsychotic medication	No	105 (89.0)	9 (7.6)	4 (3.4)	Ref	Ref
	Yes	1938 (90.0)	132 (6.1)	84 (3.9)	1.10 (0.58 to 1.92)	0.87 (0.41 to 1.86)

Participant demographics		Likely to recommend	Neutral to recommend	Unlikely to recommend	Unadjusted OR (likely vs neutral/unlikely)	Adjusted† OR (likely vs neutral/unlikely)
Age, years	<19	83 (84.7)	8 (8.2)	7 (7.1)	0.79 (0.49 to 1.49)	0.76 (0.37 to 1.55)
	19–25	569 (87.4)	46 (7.1)	36 (5.5)	Ref	Ref
	26–35	576 (90.0)	38 (5.9)	26 (5.5)	1.30 (0.92 to 1.85)	1.48 (0.96 to 2.27)
	36–50	467 (91.4)	28 (5.5)	16 (3.1)	1.54 (1.05 to 2.28)^*^	2.15 (1.31 to 3.52)**
	>50	317 (93.5)	17 (5.0)	5 (1.5)	2.11 (1.32 to 3.52)**	3.67 (1.94 to 6.97)***
Ethnicity	White	1324 (89.1)	100 (6.7)	62 (4.2)	Ref	Ref
	Black/black British	216 (93.1)	10 (4.3)	6 (2.6)	1.65 (1.00 to 2.92)	3.16 (1.51 to 6.63)**
	Asian/Asian British	206 (91.9)	12 (5.4)	6 (2.7)	1.40 (0.87 to 2.41)	1.76 (0.92 to 3.38)
	Mixed	100 (89.3)	4 (3.6)	8 (7.1)	0.98 (0.55 to 1.92)	1.40 (0.64 to 3.06)
	Other	146 (88.5)	12 (7.3)	7 (4.2)	0.94 (0.58 to 1.61)	0.99 (0.54 to 1.83)
Gender	Male	1010 (89.1)	74 (6.5)	49 (4.4)	Ref	Ref
	Female	962 (91.1)	57 (5.4)	37 (3.5)	1.25 (0.94 to 1.66)	1.04 (0.73 to 1.48)
	Other	11 (61.1)	3 (16.7)	4 (22.2)	0.18 (0.07 to 0.50)***	0.16 (0.05 to 0.52)**

*p<0.05, **p<0.01, ***p<0.001 for differences compared with the reference group.

†Adjusted for age, gender, ethnicity, duration of care, treatments offered (CBTp/family therapy/carer intervention/antipsychotic medication).

**Table 3:** Associations between clinical/demographic variables and likelihood of reporting improved mental health

Component of care		Mental health improved	Mental health unchanged	Mental health worse	Unadjusted OR (likely vs neutral/unlikely)	Adjusted† OR (likely vs neutral/unlikely)
Care coordinator case load size (mean)		17.3	17.9	17.6	0.98 (0.96 to 1.01)	0.97 (0.94 to 1.01)
Offered CBTp	No	1014 (85.6)	110 (9.2)	61 (5.2)	Ref	Ref
	Yes	1000 (92.7)	50 (4.6)	29 (2.7)	2.13 (1.61 to 2.83)***	1.95 (1.35 to 2.80)***
Offered family therapy	No	1409 (88.0)	121 (7.6)	71 (4.4)	Ref	Ref
	Yes	547 (92.1)	34 (5.7)	13 (2.2)	1.60 (1.16 to 2.25)**	1.05 (0.68 to 1.63)
Offered carer intervention	No	425 (82.5)	50 (9.7)	40 (7.8)	Ref	Ref
	Yes	1287 (92.4)	78 (5.6)	28 (2.0)	2.63 (1.95 to 3.56)***	2.49 (1.75 to 3.56)***
Offered antipsychotic medication	No	99 (79.8)	19 (15.3)	6 (4.8)	Ref	Ref
	Yes	1984 (89.7)	145 (6.6)	84 (3.8)	2.11 (1.31 to 3.28)**	1.82 (0.99 to 3.33)

Participant demographics		Mental health improved	Mental health unchanged	Mental health worse	Unadjusted OR (likely vs neutral/unlikely)	Adjusted† OR (likely vs neutral/unlikely)
Age, years	<19	89 (87.3)	10 (9.8)	3 (2.9)	0.81 (0.44 to 1.58)	1.21 (0.52 to 2.82)
	19–25	599 (89.8)	32 (4.8)	36 (5.4)	Ref	Ref
	26–35	590 (89.3)	49 (7.4)	22 (3.3)	0.97 (0.68 to 1.38)	1.15 (0.75 to 1.78)
	36–50	462 (88.0)	45 (8.6)	18 (3.4)	0.86 (0.60 to 1.24)	0.99 (0.63 to 1.56)
	>50	314 (89.5)	25 (7.1)	12 (3.4)	0.99 (0.65 to 1.52)	1.47 (0.83 to 2.57)
Ethnicity	White	1349 (89.1)	105 (6.9)	60 (4.0)	Ref	Ref
	Black/black British	222 (92.5)	10 (4.2)	8 (3.3)	1.50 (0.93 to 2.57)	1.47 (0.81 to 2.68)
	Asian/Asian British	201 (87.4)	17 (7.4)	12 (5.2)	0.84 (0.56 to 1.31)	0.85 (0.49 to 1.46)
	Mixed	104 (87.4)	11 (9.2)	4 (3.4)	0.86 (0.50 to 1.57)	0.98 (0.47 to 2.05)
	Other	157 (87.2)	17 (9.4)	6 (3.3)	0.85 (0.54 to 1.38)	0.91 (0.49 to 1.69)
Gender	Male	1029 (87.6)	96 (8.2)	50 (4.3)	Ref	Ref
	Female	981 (91.3)	56 (5.2)	38 (3.5)	1.47 (1.12 to 1.94)**	1.43 (1.02 to 2.01)^*^
	Other	16 (76.2)	4 (19.0)	1 (4.8)	0.48 (0.19 to 1.47)	0.51 (0.16 to 2.68)

*p<0.05, **p<0.01, ***p<0.001 for differences compared with the reference group.

†Adjusted for age, gender, ethnicity, duration of care, treatments offered (CBTp/ family therapy/ carer intervention/ antipsychotic medication.

Associations between exposure variables and likelihood of recommending treatment are reported in table 2 and online supplemental figure 1. Associations between demographic variables and likelihood of recommending treatment are reported in table 2 and online supplemental figure 2. Stated ORs indicate the odds that a participant with the given exposure is *more* likely (ie, likely as opposed to neutral or unlikely) to report the stated outcome.

10.1136/bmjment-2023-300716.supp1 Supplementary data

10.1136/bmjment-2023-300716.supp2 Supplementary data

People who received treatment from teams with larger care coordinator case loads were less likely to recommend the treatment they received (aOR 0.96; 95% CI 0.93 to 0.99), meaning the odds of recommending care fell by 4% on average for every one person increase in case load.

People were more likely to recommend their treatment if they had been offered CBTp (aOR 1.64; 95% CI 1.13 to 2.36), family therapy (aOR 1.72; 95% CI 1.05 to 2.82) or carer interventions (aOR 3.82; 95% CI 2.64 to 5.52). Specific demographic factors were also associated with altered likelihood to recommend treatment (people aged >35 years more likely to recommend their care than those aged 19–25 years, people with black/black British ethnicity were more likely to recommend their care than those with white ethnicity, and people with non-binary gender were less likely to recommend their care than men).

Associations between exposure variables and self-reported improvement in mental health are shown in table 3 and online supplemental figure 3. Associations between demographic variables and self-reported improvement in mental health are shown in table 3 and online supplemental figure 4. There was weak evidence that people who received treatment from teams with larger care coordinator case loads were less likely to report improved mental health (aOR 0.97; 95% CI 0.94 to 1.01—borderline significance). People were more likely to report that their mental health had improved if they had been offered CBTp (aOR 1.95; 95% CI 1.35 to 2.80) or targeted interventions for carers (aOR 2.49; 95% CI 1.75 to 3.56). There was also weak evidence that people who were offered antipsychotic medication were more likely to feel that their mental health had improved (aOR 1.82; 95% CI 0.99 to 3.33—borderline significance). Women were more likely than men to report that their mental health had improved (aOR 1.43; 95% CI 1.02 to 2.01)—no other demographic factors were significantly associated with this outcome.

10.1136/bmjment-2023-300716.supp3 Supplementary data

10.1136/bmjment-2023-300716.supp4 Supplementary data

## Discussion

This study was the first to examine relationships between components of EIP care and patient experience, and positive associations were identified for many of the core components of EIP. Those who were offered CBTp, family and carer interventions were all more likely to recommend the care they had received, and those who were offered CBTp and carer interventions were more likely to report improvements in their mental health.

Smaller care coordinator case load sizes were associated with an increased likelihood of recommending care. The advantages of smaller case loads may seem intrinsically obvious to clinicians. However, these have been difficult to demonstrate in practice, with previous studies concluding that in well-coordinated mental health services, ‘simply’ reducing case load sizes does not improve objective outcomes.^20^

However, improved experience of care (as evidenced by likelihood to recommend the care received) is an important outcome in itself. Consistent improvements in mental health or the specific symptoms of a mental disorder may not always be achievable, even with gold-standard treatments. An increased willingness to recommend the care received, on the other hand, indicates a strong therapeutic alliance between patients and professionals. Reduced care coordinator case load sizes may therefore be an important tool to facilitate personalised care and increase engagement. This may have continued benefits long after people have moved on from EIP services.^21^

Our findings substantiate existing guidelines^22^ and the recommendations made in the NCAP audit report,^16^ in that they highlight the value of a comprehensive package of evidence-based treatments in EIP. While psychosocial interventions may be considered to have a less robust evidence base for treating psychosis (than, eg, antipsychotic medication),^23^ they may have important indirect effects—improving patient experience and therefore engagement with other treatments on offer. Carer interventions in particular demonstrated strong associations with both PROMs used in this study. We would join other researchers in advocating for increased awareness of carers’ needs and efforts to provide personalised support.^24^

We also found that the likelihood of reporting positive experiences and improvements in mental health varied according to demographic factors. Some of this variation is predictable—women were more likely than men to report improvement in their mental health, consistent with established findings of worse outcomes for men both from psychotic disorders and EIP treatment specifically^25^ (likely due to disparities in other risk factors between the genders). Increasing satisfaction and perceived benefit from services with age have also been described in previous studies.^26^

However, we also found that participants from black/black British ethnic groups were more likely to recommend their care than those from white ethnic groups. This contrasts with well-documented inequalities in this area—while people from black, asian and minority ethnic (BAME) backgrounds are disproportionately affected by psychotic disorders, they are also historically more likely to experience poor outcomes and difficulties with accessing care.^27^ This noteworthy finding may suggest that EIP teams are providing a more culturally aware service offer in comparison with other settings. Alternatively, it may indicate that an increasing awareness of inequality is translating into meaningful global efforts to improve care for these groups—although we do note that BAME groups were still slightly under-represented among responders to the survey.

A less encouraging finding is the relatively low likelihood of recommending care among those who identified as transgender or a non-binary gender. While this finding should be interpreted with caution as the number of respondents in question was extremely small, it does raise the possibility that this group may face particular challenges in their treatment in this setting. This would be worthy of more targeted exploration.

Overall, the majority of people who participated in this survey would recommend the care that they received from their EIP team, and felt that their mental health improved during this period of their contact with mental health services. While many benefits resulting from EIP input have already been described, this finding alone is significant. These results compare favourably with national PROM data for other secondary care community mental health services, as well as acute and specialist mental health services.^28^ They provide further evidence of the importance of EIP care, particularly in the context of the generally poor overall outcomes experienced by people with psychotic disorders.^29^

### Strengths and limitations

This was a national study. Data were obtained from a heterogeneous sample of people recruited from almost every EIP service in England, and to our knowledge this study is the largest to date examining PROMs in this population. A variety of different models of EIP care were represented, including services that had very different care coordinator case load sizes, and that offered specific treatments to different proportions of the people under their care. The survey was designed with input from an expert group of patients and providers, and the outcomes are validated and clinically relevant. We used a principled statistical modelling approach (ie, covariates and interactions were prespecified from a priori discussions based on clinical expertise, rather than fitting models to the available data in some stepwise or data-driven way).

Several limitations should be noted. The response rate for the survey was low, and comparative data from a case note review conducted as part of the NCAP in parallel with the survey suggest that the response rate may have differed according to demographic characteristics (see table 1). It is possible that non-respondents also represented a different clinical population (eg, increased symptom burden, greater psychosocial adversity or more complex comorbidities such as substance misuse). Respondents may have had different experiences of care compared with those who did not respond. The relatively low response rate is in itself unlikely to affect the nature of the observed associations between specified exposures and self-reported outcomes. However, care should be taken in generalising these findings to the entire population of people using EIP services.

As this was an observational study, the results do not imply causal effects. It is possible that people whose care coordinator had a smaller case load, or who received specific treatments, went on to have better experiences because of these aspects of their care. However, these associations may be attributable to unmeasured confounders.

The study also relied on self-report measures and was therefore subject to people accurately recalling information (eg, about which treatments they had been offered, at a time when they may have been subject to distressing and disorienting experiences). We did not gather any information from carers or family members directly. Our primary and secondary outcome measures were both single-item PROMs, and while both are considered to have high validity for measuring patients' perceptions of care, they are limited in their ability to accurately characterise the full range of patients' experiences. Similarly, the reliability of one-off measures may be influenced by factors such as the timing of the survey in relation to the treatment received. They are unable to capture how experiences may have changed over the course of treatment, and we note that the PGI-I was validated for evaluating relatively short interventions, in comparison to EIP care which typically lasts several years.

Other methods, such as repeated measures over time, or qualitative interviews—possibly involving carers or family members in addition to patients themselves—may have allowed us to gain more detailed information about people’s experiences.

### Clinical implications

Guidelines for EIP service implementation currently include recommendations for maximum care coordinator case load sizes.^30^ These emerged despite a lack of clear evidence about whether reduced case load sizes translate to improved outcomes. However, this study highlights the importance of reduced case loads for patient experience. Our results also reinforce the importance of a comprehensive package of varied treatments to optimise EIP care. CBTp and carer interventions appear particularly valuable for patients’ mental health.

Our results also identify groups at increased risk of poor experience from EIP treatment. Some of these risk factors are established in existing literature (younger patients, male gender), while some (transgender or non-binary gender) require further exploration. On a positive note, our study also provides some indication of improvements in historical inequalities (improved experience of care in black/black British ethnic groups), or at least suggests that these are being addressed as part of an EIP model of care.

We would reiterate recommendations made following previous research in this area^9^—that EIP teams employ PROMs as part of routine service evaluation, in order to organise care around patients’ specific experiences, preferences and needs. We note again the low response rate to the survey used in this study, and the possibility that people with certain demographic or clinical characteristics may be less likely to participate in this form of service evaluation. Targeted research such as qualitative studies of EIP patients may be helpful to identify and address barriers to participation, in order to ensure that a full range of perspectives are captured in other similar studies in the future.

More research is needed to optimise EIP service delivery. This may be best achieved through large-scale prospective observational studies of patients attending differently structured services, where different components of care are available. Such studies may confirm our observed associations between components of EIP care and patient experience and expand on these by examining the differential impact on a wider range of treatment outcomes. Ideally, these would inform the development of a predictive model and gold-standard treatment package to optimise outcomes for people with psychosis.

## Data Availability

Data are available upon reasonable request. Data may be obtained from a third party and are not publicly available. RW, AM and AQ had access to the full study data set. The data are not publicly available but are held by the NCAP team at the College Centre for Quality Improvement, Royal College of Psychiatrists, and could be made available upon reasonable request.
