# Supplementary figures and images for: Predictors of positive patient-reported outcomes from ‘Early Intervention in Psychosis’: a national cross-sectional study
Source: BMJ Ment Health. 2023 Aug 4;26(1):e300716. doi: 10.1136/bmjment-2023-300716 (PMC10577709; doi:10.1136/bmjment-2023-300716)

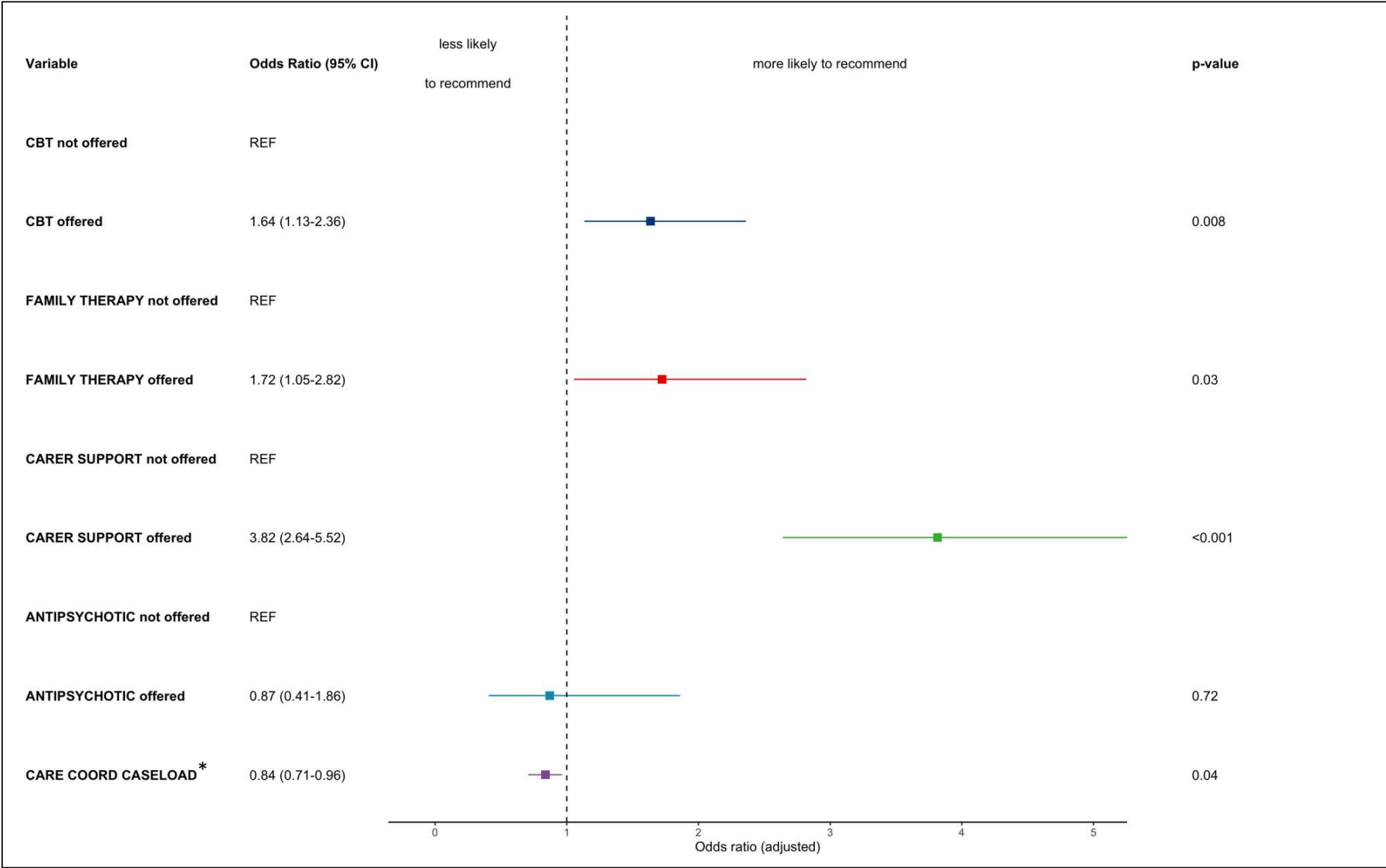

\*plotted odds ratio for 5 unit increase in care coordinator caseload

Supplement: Supplementary data [file bmjment-2023-300716supp001.pdf]

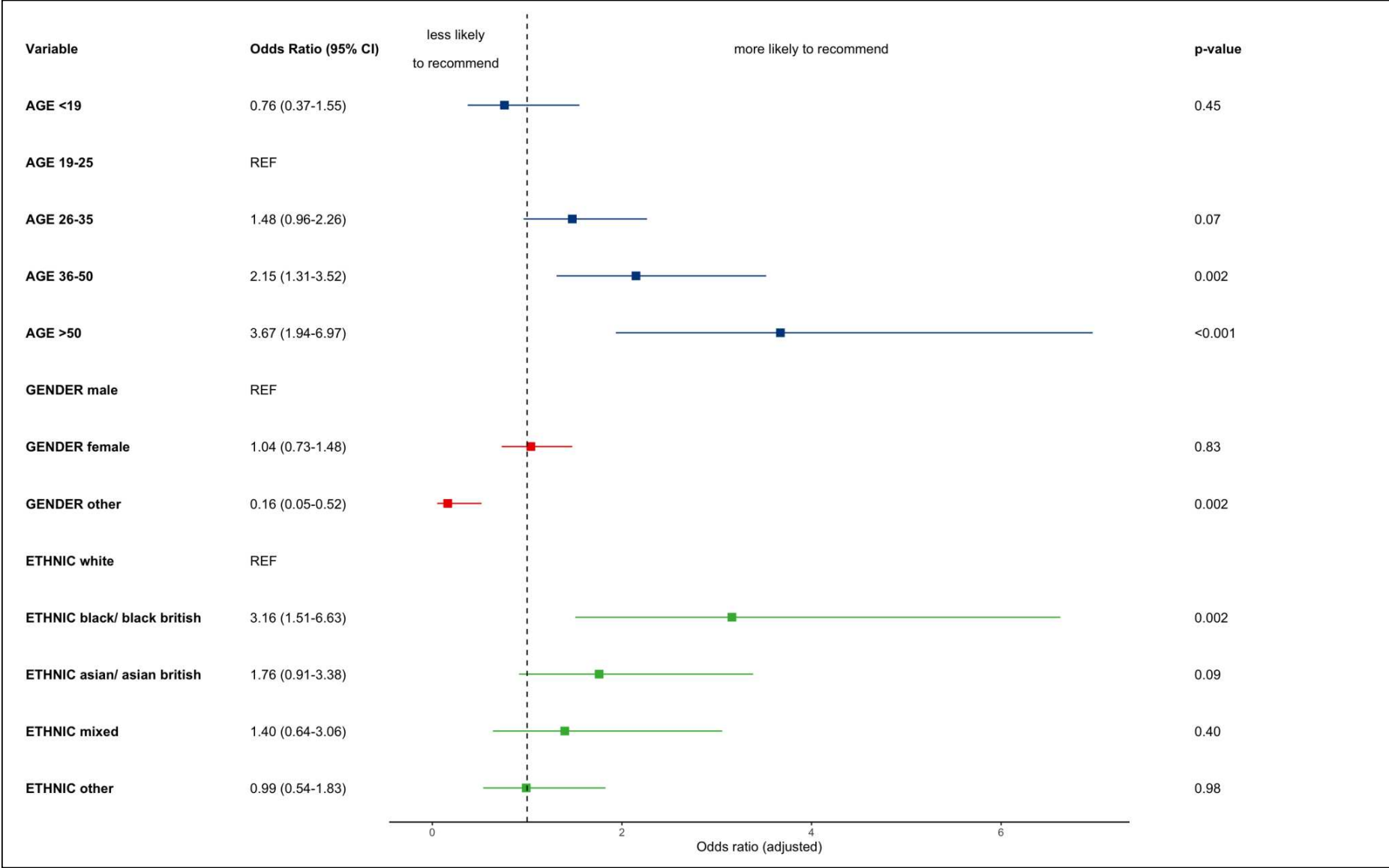

Supplement: Supplementary data [file bmjment-2023-300716supp002.pdf]

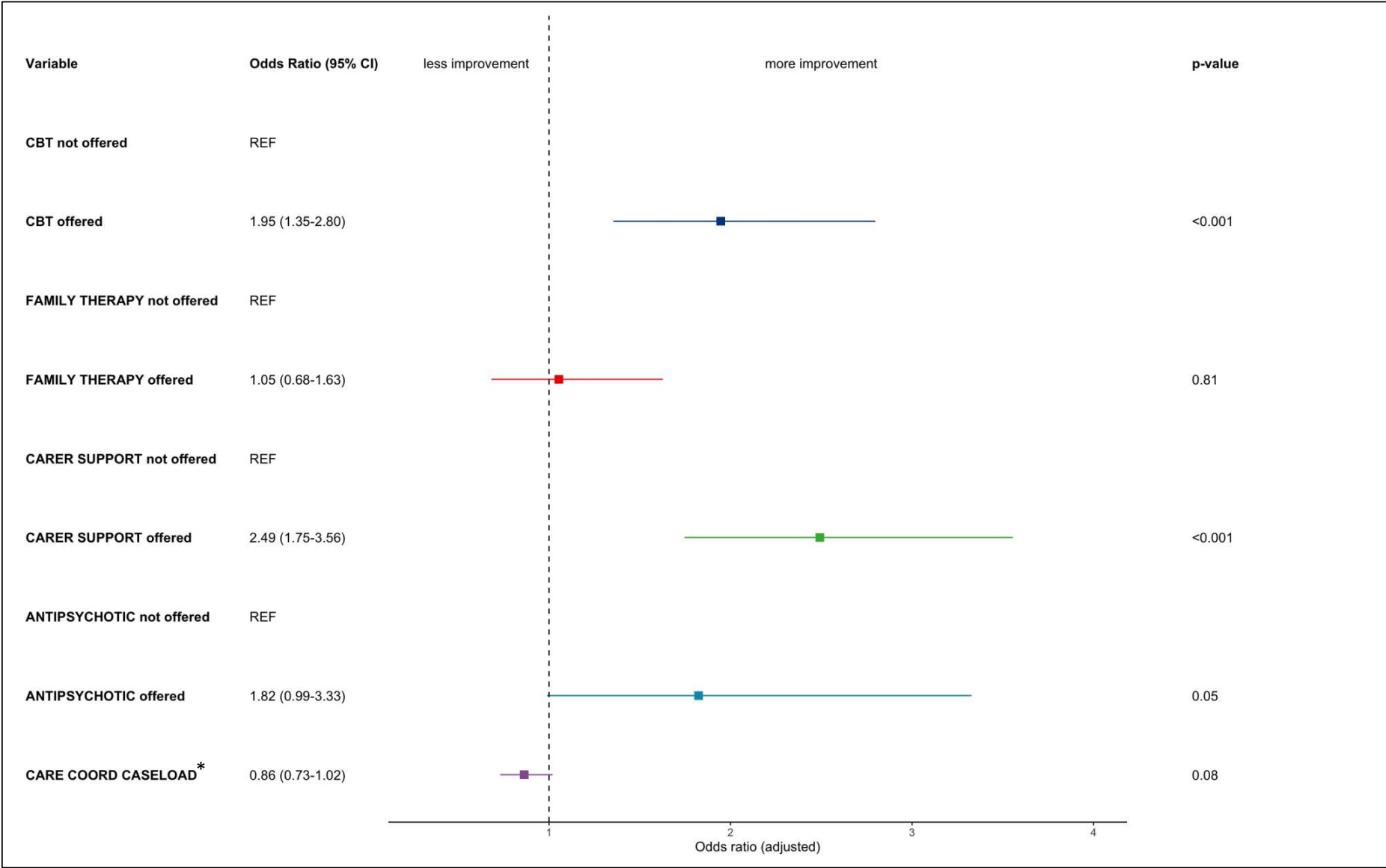

\*plotted odds ratio for 5 unit increase in care coordinator caseload

Supplement: Supplementary data [file bmjment-2023-300716supp003.pdf]

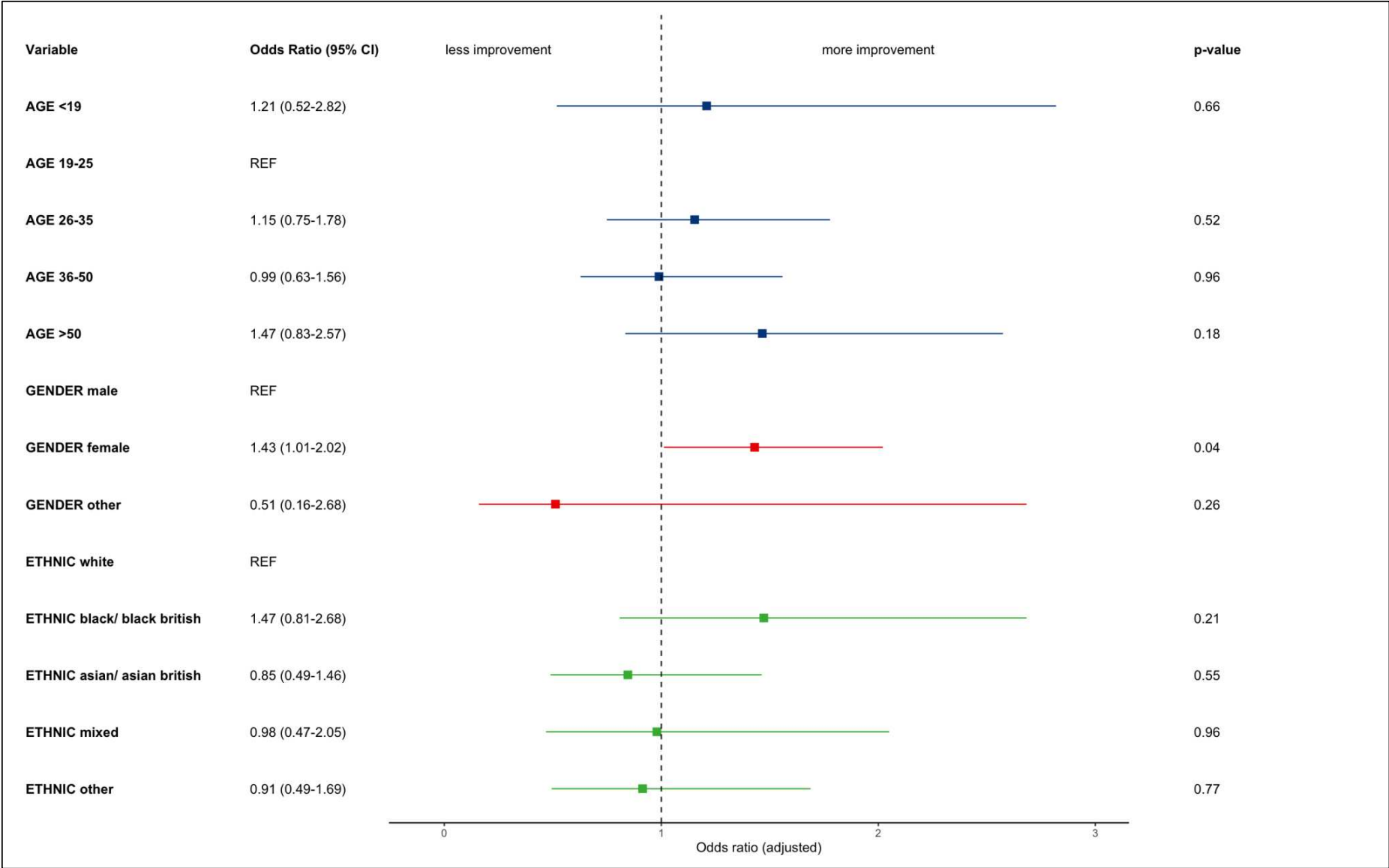

Supplement: Supplementary data [file bmjment-2023-300716supp004.pdf]
